# Clinical Outcome of Cannulated Screw Fixation with Suture Tape Augmentation in Geriatric Transverse Patellar Fracture – A Case Series

**DOI:** 10.5704/MOJ.2511.007

**Published:** 2025-11

**Authors:** MA Muhamed-Fuad, AH Ab-Halim

**Affiliations:** Department of Orthopaedics, Universiti Putra Malaysia, Serdang, Malaysia

**Keywords:** knee injuries, patella, bone screws, suture tape, aged

## Abstract

**Introduction:**

Patellar fractures were common and often challenging to manage, particularly in the elderly population. Achieving stable anatomic reduction and maintaining it throughout the perioperative period was crucial for restoring knee function, especially in patients with osteopenic bone.

**Material and Methods:**

This was a retrospective case series of geriatric patients who presented to our centre with closed transverse patellar fractures between 2022 and 2023. All fractures were classified as AO 34C1 under the Arbeitsgemeinschaft für Osteosynthesefragen (AO) classification and were considered fragility fractures, having resulted from a fall from standing height. Only patients aged over 60 years were included. All underwent open reduction and internal fixation using cannulated screws with suture tape augmentation. The Knee Society Score (KSS) was evaluated at four months post-operatively, and patients were followed for complications for up to one year.

**Results:**

Six cases met the inclusion criteria. The mean displacement was 19mm, and the average operative time was 45.17 minutes. The average time to radiographic union was 8.67 weeks. At a mean follow-up of 12.8 months, none of the patients required revision surgery or hardware removal. No patient reported issues related to implant prominence or pain during kneeling. The KSS at four months ranged from 87 to 97, with a mean score of 91.

**Conclusion:**

Cannulated screw fixation with suture tape augmentation appeared to be a safe and effective method for treating transverse patellar fractures in the elderly population, offering stable fixation and favourable functional outcomes.

## Introduction

Patellar fractures were relatively common, accounting for approximately 1% of all skeletal injuries. Achieving and maintaining a stable anatomic reduction during the perioperative period was essential for restoring knee function, particularly in elderly patients with osteopenic bone^1^. Several fixation techniques had been described, including cerclage compression wiring (previously referred to as tension band wiring), cannulated screw fixation, and plate fixation. Cerclage compression wiring was widely used but presented challenges in cases involving comminuted fractures or poor bone quality. Its failure rate had been well-documented in the literature. Although plate fixation provided more rigid stability, its application was often difficult due to the patella’s subcutaneous position and the associated risk of soft tissue irritation.

More recently, the use of cannulated screws with suture tape augmentation had been introduced as a promising alternative for improving fixation strength in osteoporotic bone^2^.

## Materials and Methods

This was a retrospective case series of geriatric patients who presented to our centre with closed transverse patellar fractures between 2022 and 2023. Patients were included if they met the criteria for fragility fractures, defined as a fall from standing height and age over 60 years, and were classified as AO 34C1 based on the Arbeitsgemeinschaft für Osteosynthesefragen (AO) classification. Fracture displacement was measured on lateral radiographs, using the distance at the anterior cortex.

All patients that underwent open reduction and internal fixation using cannulated screws with suture tape augmentation were included. They were evaluated for radiographic union time and Knee Society Score (KSS) at four months post-operatively and monitored for complications for up to one year. The KSS was assessed at four months, during their final specialised clinic visit, before patients were transferred to general orthopaedic follow-up for long-term monitoring.

A standard midline longitudinal incision was made over the patella. The fracture site was exposed and anatomically reduced using a malleolar clamp. Two guidewires for 4.0mm cannulated screws were inserted from the inferior pole to the superior pole of the patella, ensuring both wires were parallel and centrally positioned.

**Fig. 1:**
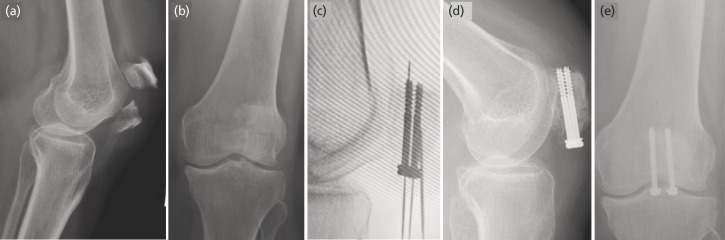
(a) and (b) plain radiograph show transverse patellar fracture (Classification; AO 34C1). (c) Cannulated screw fixation inserted with the guidance of guide wire and image intensifier. (d) and (e) radiographic union over fracture were evaluated during follow-up.

The tip of each guidewire was advanced to the far cortex, and screw length was measured. The wires were then extended into the quadriceps tendon, with minimal soft tissue dissection to aid identification of the suture passer later in the procedure. A cannulated drill bit was used to prepare the path, ensuring penetration through the second cortex. Half-threaded 4.0mm stainless steel cannulated screws with washers were then inserted for fixation in all cases (Fig. 1).

**Fig. 2:**
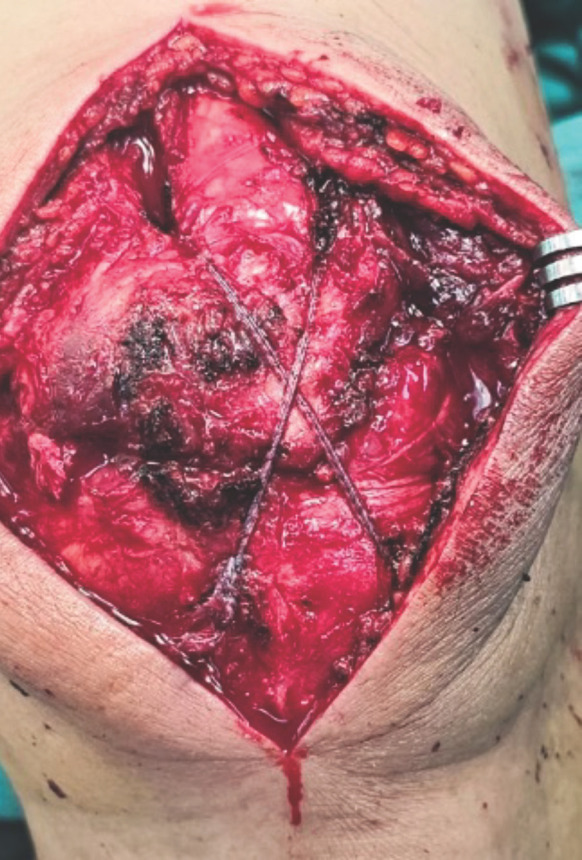
Fiber suture tape was tightened in horizontal figure-of-eight manner.

The screw threads passed across the fracture line and engaged the far cortex. A 2.0mm suture tape was passed through the cannulated screws using a wire passer and retrieved through the previously dissected area. The suture tape was secured in a horizontal figure-of-eight configuration (Fig. 2).

After fixation, the knee was further manipulated into flexion up to 110° and full extension to assess for any displacement and to evaluate the stability of the construct. The retinaculum was repaired, and the wound was closed in layers. The operative time (skin-to-skin) was recorded. Post-operatively, all patients were protected with a cylinder slab or knee brace for two weeks to allow for wound healing and to minimise the risk of falls during early ambulation training. Weight-bearing was permitted as tolerated. Full range of motion exercises were initiated at two weeks post-operatively after removal of slab or knee brace. Follow-up assessments were conducted at two weeks for wound inspection and brace removal. Patients were then reviewed every four to six weeks to monitor fracture union and range of motion. At four months, a specialised clinic visit was scheduled for Knee Society Score (KSS) assessment. A final follow-up was conducted at around one year follow-up under general clinic to evaluate for any complications before discharge from care.

## Results

A total of 21 patients aged over 60 years sustained closed patellar fractures at our centre during the study period. However, only six patients met the inclusion criteria and were enrolled in the study. The remaining patients were excluded due to comminuted fracture patterns, surgeon preference for conventional techniques, non-displaced fractures managed conservatively, or financial constraints.

The mean displacement at the anterior cortex between the proximal and distal patellar fragments was 19mm. All six included fractures healed without complications such as infection, non-union, or hardware failure during the one-year follow-up period (Table I). The mean operative time was 45.17 minutes. Radiographic union was achieved between 8 and 10 weeks, with a mean union time of 8.67 weeks.

**Table I:** Patient data on operation time, patellar displacement, union time, complication, ROM knee, duration of follow-up and KSS at four months.

Patient	A	B	C	D	E	F
Age (years old)	64	68	70	73	65	68
Gender	male	male	female	male	female	male
Patellar fracture displacement (mm)	22	20	18	16	18	20
Operation time (min)	40	50	36	39	38	40
Union time (weeks)	8	10	8	8	8	10
Complications at one year follow-up	nil	nil	nil	nil	nil	nil
ROM knee at four months	0–110	5–100	0–105	0–110	0–110	0–105
Follow-up (months)	12	13	14	13	12	13
KSS four months	92	87	95	96	97	91

None of the patients reported implant-related discomfort or pain during kneeling. Most patients regained more than 100° of knee flexion. Two patients (B and F) experienced mild difficulty performing full squats but did not report problems rising from a seated position or performing daily activities. Patient B had a delayed surgery due to multiple comorbidities, requiring two weeks of pre-operative optimisation. The remaining patients underwent surgery within one week of injury through the fragility fracture operative pathway.

None of the patients were employed at the time of treatment; however, all were able to attend follow-up appointments independently, return to their daily activities and ambulated without walking aids. The Knee Society Score (KSS) at four months ranged from 87 to 97, with a mean of 91. All patients completed follow-up up to one year, with a mean follow-up duration of 12.8 months. Pre-operative KSS scores were not collected due to the acute nature of injury management.

## Discussion

Patellar fractures, which represented approximately 1% of all skeletal injuries, posed significant challenges, particularly in elderly patients^3^. The reduced bone quality in this population required robust fixation techniques to ensure proper healing and functional recovery. Our case series of six elderly patients treated with cannulated screw fixation augmented by suture tape demonstrated the potential of this technique in achieving favourable clinical outcomes.

Cerclage compression wiring, previously known as tension band wiring, was a widely adopted technique. However, it presented several limitations, especially in cases involving comminuted fractures or osteoporotic bone. This method was associated with complications such as loss of reduction, implant failure, and the frequent need for hardware removal^4-6^. While plate fixation offered more rigid stabilisation, its use was often limited by the patella’s subcutaneous location and the associated risks of soft tissue irritation and infection^1^.

A multicentre retrospective study by Oyama *et al*^7^ identified several predictors of complications following cerclage compression wiring. Their findings showed that failure to bend both ends of the Kirschner wires significantly increased the risk of implant migration. Furthermore, a larger patella-to-wire ratio and wider spacing between K-wires were also linked to implant breakage. Interestingly, they found no significant factors associated with loss of reduction. These results highlighted the importance of meticulous surgical technique, particularly the proper bending of wire ends and precise placement of cerclage components, to minimise complications.

Although conservative treatment may be sufficient for some non-displaced fractures, surgical fixation is generally recommended for displaced or comminuted patterns^8^. The choice of fixation method often depends on fracture morphology, bone quality, and the surgeon's experience or preference. Traditional techniques such as cerclage compression wiring, while widely practiced, are known to carry risks including wire migration, breakage, and hardware-related pain, often necessitating re-operation in up to 4.2% of cases^6,7^.

A modified version of cerclage compression wiring using cannulated screws has gained popularity in recent years^2^. However, achieving and maintaining anatomic reduction remains challenging in more complex fracture patterns. Plate fixation may offer improved mechanical strength, but its use is technically demanding due to the superficial location of the patella^1^. In cases of fixation failure, Xue *et al* proposed a two-tension-band technique as a valuable salvage procedure^9^.

Our approach, utilising cannulated screws with suture tape augmentation was designed to enhance fixation strength while minimising hardware-related complications in elderly patients with transverse patellar fractures. The suture tape cerclage method described by Monaco *et al* provided additional support and potentially reduced the risk of fragment displacement, especially in osteoporotic bone^10^. Our technique closely resembled that described by Posner *et al*, though we did not employ a formal cerclage tensioning device due to unavailability. Posner’s study concluded that this method was effective and reproducible, while also reducing hardware-related symptoms and re-operation rates^11^.

Biomechanical studies have shown that cannulated screws provide greater stability and higher load-to-failure thresholds compared to traditional K-wire techniques. K-wires are also more likely to be prominent and migrate, which increases the likelihood of symptomatic hardware and the need for removal^12-16^. Monaco *et al*^10^ also cautioned that if the screw tip extends too far beyond the far cortex, it may abrade the suture tape. The use of blunt-tipped cannulated screws where available could be an interesting area for future investigation.

Post-operative immobilisation was not extensively discussed in earlier studies, but in our series, it was applied temporarily to protect the repair from unexpected fall and facilitate early weight-bearing ambulation training. By the second week, after wound healing and improved patient confidence in ambulation, immobilisation was discontinued to allow commencement of range-of-motion exercises.

Our results showed uncomplicated healing, restored mobility, and satisfactory functional outcomes in all six patients. No complications were observed, and the KSS at four months reflected positive short-term recovery. These findings suggest that cannulated screw fixation with suture tape augmentation may offer a reliable and effective alternative for treating transverse patellar fractures in elderly patients. However, larger studies with longer-term follow-up are needed to validate these results and compare this method directly with other fixation techniques. Further research is also encouraged to evaluate the biomechanical advantages of suture tape augmentation and its long-term influence on fracture healing.

## Conclusion

Cannulated screw fixation with suture tape augmentation is a promising technique for managing patellar fractures in elderly patients. This approach achieves stable fixation, promotes healing, and restores function while minimising complications especially in osteopenia bone.
